# Intra-Articular Local Anesthetics in Temporomandibular Disorders: A Systematic Review and Meta-Analysis

**DOI:** 10.3390/jcm13010106

**Published:** 2023-12-24

**Authors:** Karolina Lubecka, Kamila Chęcińska, Filip Bliźniak, Maciej Chęciński, Natalia Turosz, Adam Michcik, Dariusz Chlubek, Maciej Sikora

**Affiliations:** 1 Department of Oral Surgery, Preventive Medicine Center, Komorowskiego 12, 30-106 Cracow, Poland; lubeckarolina@gmail.com (K.L.); fblizniak@gmail.com (F.B.); maciej@checinscy.pl (M.C.); 2 Department of Glass Technology and Amorphous Coatings, Faculty of Materials Science and Ceramics, AGH University of Science and Technology, Mickiewicza 30, 30-059 Cracow, Poland; checinska@agh.edu.pl; 3 Institute of Public Health, Jagiellonian University Medical College, Skawińska 8, 31-066 Cracow, Poland; natalia.turosz@gmail.com; 4 Department of Maxillofacial Surgery, Medical University of Gdansk, Mariana Smoluchowskiego 17, 80-214 Gdańsk, Poland; adammichcik@gumed.edu.pl; 5 Department of Biochemistry and Medical Chemistry, Pomeranian Medical University, Powstańców Wielkopolskich 72, 70-111 Szczecin, Poland; sikora-maciej@wp.pl; 6 Department of Maxillofacial Surgery, Hospital of the Ministry of Interior, Wojska Polskiego 51, 25-375 Kielce, Poland

**Keywords:** temporomandibular joint disorders, intra-articular injections, bupivacaine, lidocaine, articaine, mepivacaine

## Abstract

This systematic review with meta-analysis was conducted to evaluate the effectiveness of local anesthetic administration into temporomandibular joint cavities in relieving pain and increasing mandibular mobility. Randomized controlled trials were included with no limitation on report publication dates. Final searches were performed on 15 October 2023, using engines provided by the US National Library, Bielefeld University, and Elsevier Publishing House. The risk of bias was assessed using the Cochrane Risk of Bias 2 tool. Articular pain and mandible abduction values and their mean differences were summarized in tables and graphs. Eight studies on a total of 252 patients evaluating intra-articular administration of articaine, bupivacaine, lidocaine, and mepivacaine were included in the systematic review. None of the eligible studies presented a high risk of bias in any of the assessed domains. An analgesic effect of intra-articular bupivacaine was observed for up to 24 h. In the long-term follow-up, there were no statistically significant changes in quantified pain compared to both the baseline value and the placebo group, regardless of the anesthetic used (articaine, bupivacaine, and lidocaine). There is no scientific evidence on the effect of intra-articular administration of local anesthesia on the range of motion of the mandible. Therefore, in the current state of knowledge, the administration of local anesthetics into the temporomandibular joint cavities can only be considered as a short-term pain relief measure.

## 1. Introduction

### 1.1. Background

The pain associated with mastication results mainly from abnormal function of the temporomandibular joints or masticatory muscles [1,2,3,4]. In the course of physical examination, it is possible to distinguish muscle pain from joint pain, which guides further diagnosis and treatment [5,6,7]. The severity of articular pain is measured primarily on a visual analog scale [8,9]. Painful reduction of jaw mobility, and thus difficulty with food intake, is a significant factor in deteriorating the patient’s quality of life [10,11,12,13,14]. In cases of severe articular pain with limited mouth opening, the main cause may be difficult to determine, and the therapy undertaken is sometimes empirical [1,15]. The range of methods for treating articular pain and limited mandibular mobility is very wide and combination therapies are often used [3,4,5,6,7,10]. Depending on the specific diagnosis and the severity of the symptoms, psychotherapy, physiotherapy, systemic pharmacotherapy, splint therapy, dry needling and intramuscular injections, intra-articular injections, arthrocentesis, arthroscopy, open joint surgery, and joint replacement are used [3,4,5,6,7,10]. Due to their minimal invasiveness, intra-articular administration of drugs, hyaluronic acid, and blood products are the subject of current scientific research [5,10]. Too slow or ineffective treatment of pain induces the search for ad hoc relief. One of the obvious solutions to relieving persistent pain is to start drug therapy [16]. An alternative to systemic analgesic pharmacotherapy is the local administration of drugs. The professional literature indicates the possibility of performing nearby nerve blocks, intra-articular administration of anti-inflammatory drugs (corticosteroids and nonsteroidal anti-inflammatory drugs), analgesics (opioids), or local anesthetics [17,18,19,20,21,22].

### 1.2. Rationale

Local anesthetics are well-researched, inexpensive, widely used, and easily available [23,24]. Intra-articular administration of local anesthetics is one of the recognized, albeit controversial, orthopedic procedures [25,26]. According to the latest reports, cytotoxicity of bupivacaine in intra-articular injections is suspected, and there have been many reports of chondrolysis after shoulder arthroscopy in which intra-articular injections of anesthetics were used [25,27]. Injection of local anesthetics into the temporomandibular joint cavities is not commonly performed [21,28]. The growing number of scientific publications on this topic allows for a first cross-sectional evaluation and encourages a critical assessment of the effectiveness of the discussed therapy [21,28]. 

### 1.3. Objectives

The primary objective of this systematic review with meta-analysis is to compare the effectiveness of local anesthetic administration in temporomandibular joint cavities in relieving articular pain compared to placebo or other substances. An analogous comparison with regard to the change in the range of mandibular mobility was adopted as a secondary objective.

## 2. Materials and Methods

This study was conducted in accordance with the Preferred Reporting Items for Systematic Reviews and Meta-Analyses guidelines and reported in the International Prospective Register of Systematic Reviews database under the number: CRD42023484735 [29,30].

### 2.1. Eligibility Criteria

The review included randomized controlled trials of the injection of local anesthetics into the temporomandibular joints. The inclusion of studies on healthy volunteers was intended to ensure the comprehensiveness of the systematic review. The outcome criterion was taken into account when qualifying for the meta-analysis, but failure to meet it did not exclude the report from inclusion in the systematic review. Data on changes in articular pain intensity or mandible mobility were required. No time frame limits were applied. Details are presented in Table 1.

### 2.2. Information Sources

This systematic review was conducted using three of the leading medical database search engines: (1) the US National Library of Medicine PubMed, (2) the German Bielefeld Academic Search Engine, and (3) the Dutch Elsevier Scopus [31,32,33]. All final searches were conducted on the same day, 15 October 2023.

### 2.3. Search Strategy

The search strategy was based on the Problem and Intervention eligibility criteria. It was implemented in the form of a single query, common to all search engines: “temporomandibular joint” AND (injection OR injections) AND (“local anesthetic” OR “local anaesthetic” OR benzocaine OR procaine OR chloroprocaine OR lidocaine OR prilocaine OR tetracaine OR bupivacaine OR cinchocaine OR ropivacaine).

### 2.4. Selection Process

Records identified during the medical database search were transferred to the Rayyan automation tool (Qatar Computing Research Institute, Doha, Qatar and Rayyan Systems, Cambridge, MA, USA) [34]. This tool identified potential duplicates, which were manually verified and, if confirmed, removed (M.C. and K.C.). Then, continuing the use of Rayyan, the same researchers performed a screening based on titles and abstracts. Records identified unanimously as not meeting the Problem or Intervention criteria were discarded. In cases of discrepancies in the assessment, a given record was left for full-text verification of the report. Eligibility determined on the basis of the full content of the reports was initially assessed by two researchers (M.C. and K.C.) and, in case of doubt, discussed among the entire team until consensus was reached.

### 2.5. Data Collection Process

The data needed for synthesis were extracted (M.C. and K.C.) without the use of automation tools, based only on the content of the reports. The data collected from the content of the reports were initially entered into a summary table, and its refined version was placed in the Results section of this paper.

### 2.6. Data Items

The researchers collected the following data items from the content of primary study reports: (1) initial severity of articular pain; (2) final severity of articular pain; (3) initial range of mandible abduction; and (4) final range of mandible abduction. Joint pain expressed on a visual analog scale (VAS) was preferred, and in the absence of this variable, a numerical rating scale (NRS) was accepted and converted proportionally to 0–10 if necessary. The quantified pain values were unified on a scale of 0–10. For several different measurements of mandible abduction range, preference was given in the following order: (1) maximum unassisted mouth opening; (2) maximum mouth opening without pain; and (3) maximum manually assisted mouth opening. These data were collected for both the study and control groups.

Additionally, the following data were extracted: (1) first author and year of publication of the report; (2) the number of patients in the study and control groups; (3) type of local anesthetic used in the study group; (4) dose of the single-administered preparation; (5) number of injections and interval between injections; (6) interventions in control groups; and (7) description of co-interventions in study and control groups.

### 2.7. Study Risk of Bias Assessment

A bias risk assessment was performed (K.L. and F.B.) using the revised Cochrane risk-of-bias tool for randomized trials (RoB 2) and visualized using the Robvis tool (c7c1bdd) [35,36].

### 2.8. Effect Measures

For the purposes of synthesizing and presenting the results, mean differences were calculated for (1) articular pain and (2) the range of mandible abduction. A MedCalc tool was used (MedCalc Software (22.016), Ostend, Belgium) [37].

### 2.9. Synthesis Methods

All studies with a risk of bias lower than high were qualified for synthesis. Synthesis was performed by combining data extracted from reports and mean difference results in a summary table. The synthesis results were presented in charts using Google Workspace tools (Google LLC, Mountain View, CA, USA, (Version: 16 October 2023 Scheduled Release)).

### 2.10. Reporting Bias Assessment

In the case of missing data, this fact was noted, but the series was not discarded. No further reporting bias assessments were undertaken.

### 2.11. Certainty Assessment

The summary of findings was tabulated with the risk of bias in the source reports provided.

## 3. Results

### 3.1. Study Selection

**Figure 1:**
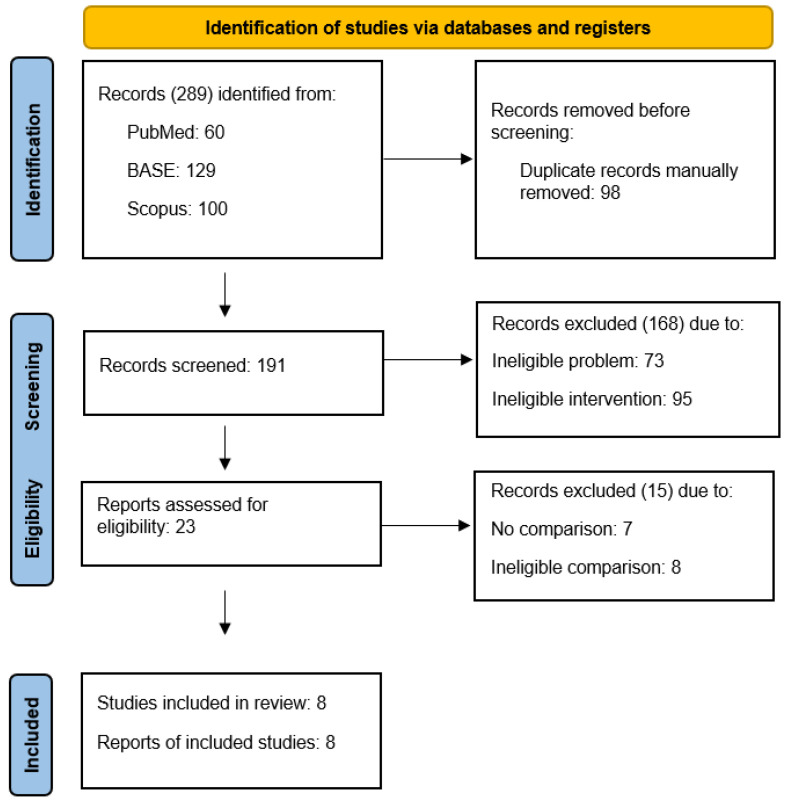
PRISMA flow diagram.

The selection process identified 23 reports describing the administration of local anesthetics into the temporomandibular joint cavities. Of these, 15 were excluded from the full-text review due to the lack of a control group or the inability to assess the effect of local anesthetics despite control groups present (Table 2) [38,39,40,41,42,43,44,45,46,47,48,49,50,51,52]. Non-qualified reports are addressed in the Discussion section. Eight reports presented randomized trials comparing interventions differing only in the intra-articular administration of local anesthetic (Table 3) [53,54,55,56,57,58,59,60]. The data from their content were extracted, synthesized, and analyzed in the Results section of this review. The detailed selection process is illustrated in a flow diagram (Figure 1).

### 3.2. Study Characteristics

Eight eligible randomized clinical trials were conducted on a total of 252 patients [53,54,55,56,57,58,59,60]. Articaine (4%, one study), bupivacaine (0.25–0.5%, three studies), lidocaine (0.2–2%, two studies), and mepivacaine (1–3%, two studies) were used. In the study groups, 1 to 3 or as-needed interventions were performed at intervals of 2 to 42 days, which gave a total dose of anesthetic from 2.5 to 40 mg. Six of the studies reported results for placebo groups (Table 4) [54,55,56,57,58,59].

### 3.3. Risk of Bias in Studies

**Figure 2:**
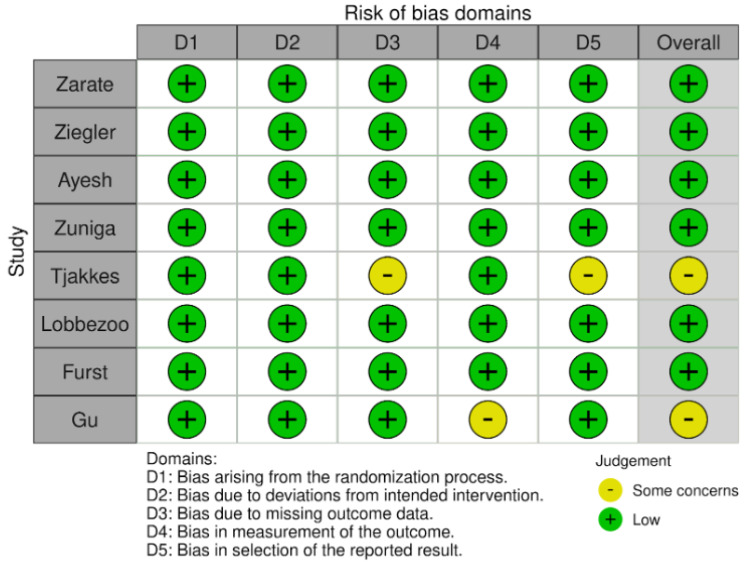
Risk of bias in studies: traffic light plot.

**Figure 3:**
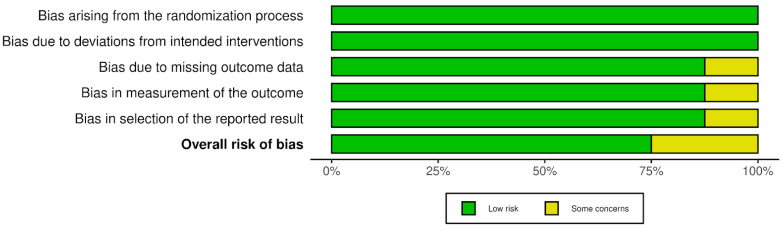
Risk of bias in studies: summary plot.

The risk of bias results obtained using the Cochrane risk-of-bias tool for randomized trials (RoB 2) are illustrated in Figure 2 and Figure 3. None of the studies identified any risks related to the randomization process or deviations from the intended interventions. There were some concerns in the remaining domains but no high risk of bias was noted. Therefore, none of the studies were rejected at this stage.

### 3.4. Results of Individual Studies

#### 3.4.1. Pain Intensity

The initial values of articular pain in the study and placebo groups and their change over time are presented in Table 5. The qualified reports by Zuniga et al. and Gu et al. described the change in the severity of articular pain, but the results could not be quantified [56,60]. In the study by Ziegler et al., the intervention was performed three times; only the initial pain values and those during the observation period after the first and before the second intervention were entered into the table. Due to the lack of standard deviations provided, the standard error of the calculated mean differences could not be determined for the study by Ziegler et al. [54]. In the study by Ayesh et al., partial pain values were not recorded, which made further processing of the results impossible [55]. In the studies by Zarate et al. and Tjakkes et al., statistically significant differences in articular pain values compared to the initial values were observed over a period of 2 weeks to a year [53,57]. Tjakkes et al. reported numerical results of changes in the intensity of joint pain for the group receiving a local anesthetic versus the placebo group. Fourteen days after the intervention, articular pain decreased in the treated group and increased in the control group. The difference in mean was −1.0 ± 0.9. However, this difference was not statistically significant (*p* = 0.28). Pain severity during this period was lower at every measurement for the local anesthetic groups [57]. Furst et al. reported only post-intervention pain values. In the study by these authors, no statistically significant differences in pain intensity were observed in any of the patient groups in the period from 4 to 24 h after the intervention [59].

#### 3.4.2. Mandibular Abduction

The range of mandibular abduction was measured only in some of the studies included in this review. Zarate et al. examined the maximum pain-free mandibular abduction, which they defined as the interincisal distance at the opening to the point of discomfort [53]. Tjakkes et al. examined maximum unassisted mandibular abduction, which they defined as the interincisal distance with maximum mouth opening on request [57]. Lobbezzo et al. took this parameter into account but as constant values for which they determined the tension of the masticatory muscles [58]. The study by Gu et al. presented only unquantified results, which made further processing impossible [60]. Therefore, Table 6 presents only the results from the studies by Zarate et al. and Tjakkes et al., which, due to their paucity, cannot be further processed [53,57]. The extent of mandibular abduction after 3 months of follow-up in the study by Zarate et al. did not differ significantly (*p* = 0.59) between the lidocaine and lidocaine plus dextrose groups. In both groups, there was an increase in these values compared to the initial ones, but it was not statistically significant [53]. During a two-week follow-up period, Tjakkes et al. did not observe a statistically significant difference between the abduction gain among the patient groups (*p* = 0.10) [57].

### 3.5. Results of Syntheses

**Figure 4:**
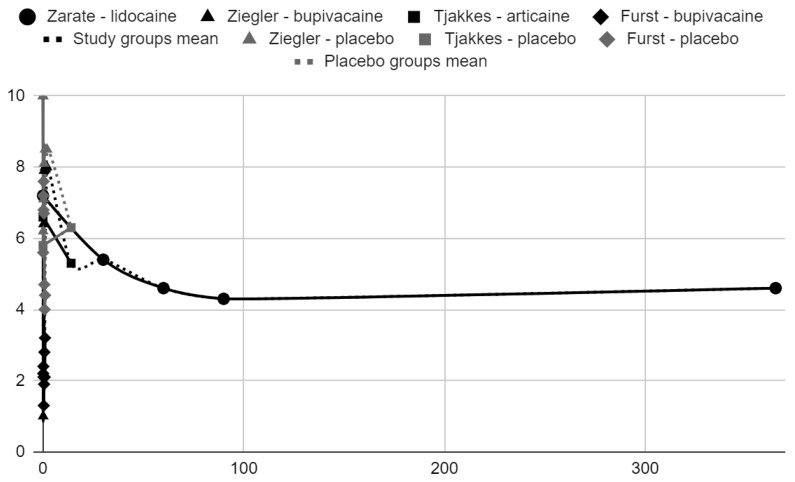
VAS/NRS pain intensity over time in days.

**Figure 5:**
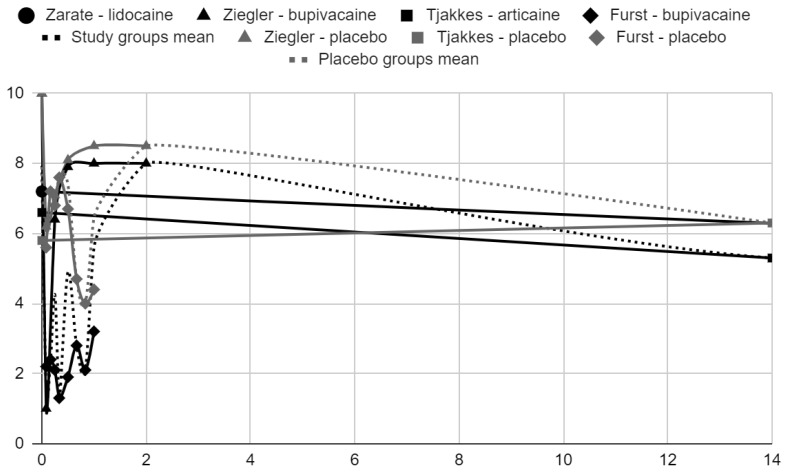
VAS pain intensity over time in days in range 0–14.

**Figure 6:**
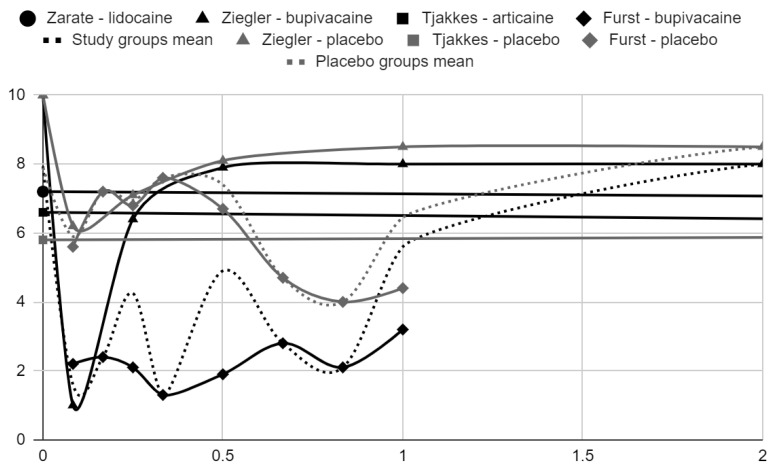
VAS pain intensity over time in days in range 0–2.

**Figure 7:**
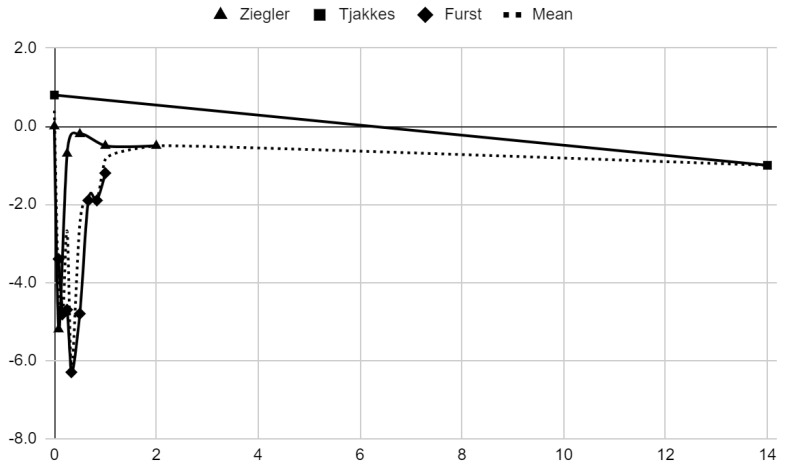
Differences in VAS pain between treatment and placebo groups over time in days.

**Figure 8:**
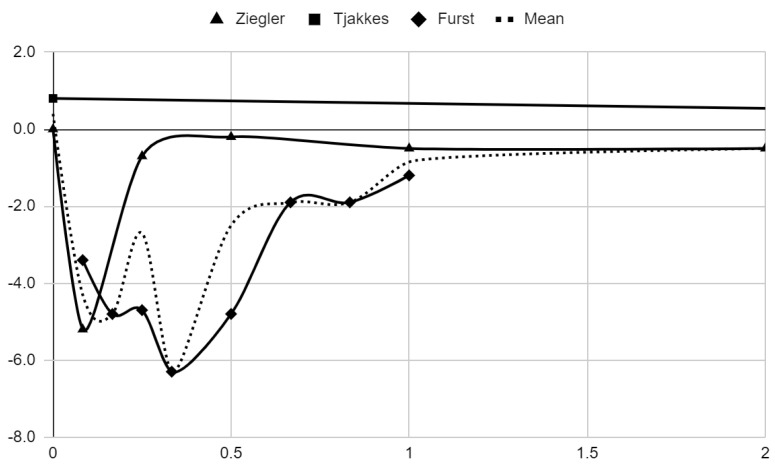
Differences in VAS pain between treatment and placebo groups over time in days in range 0–2.

Below, graphical summaries of the results of the mean values of articular pain intensity for the groups receiving anesthetics (black lines) and the placebo groups (gray lines) are presented. Due to the large number of time points for the initial observation period, the same data are illustrated multiple times but at different scales in Figure 4, Figure 5 and Figure 6. The following figures illustrate the differences in pain intensity between the study groups and placebo on two observation time scales (Figure 7 and Figure 8). Attempts to fit linear regression models were unsuccessful; therefore, mean values are illustrated (dashed lines).

### 3.6. Certainty of Evidence

The key results of this systematic review are summarized in Table 7. The articular pain results are supported by four and the mouth opening range results by two randomized clinical trials, all free of high risk of bias in any of the domains assessed.

## 4. Discussion

### 4.1. General Interpretation of the Results in the Context of Other Evidence

#### 4.1.1. Randomized Controlled Trials

The administration of local anesthetics into the cavities of the temporomandibular joints seems to be justified only in the context of temporary relief of articular pain. The use of bupivacaine appears to provide an immediate analgesic effect that lasts for up to 24 h [54,59]. This may be sufficient to transfer the patient to a higher-reference center and undergo other types of treatment. In the long-term follow-up, none of the local anesthetics resulted in statistically significant improvement in pain either from baseline or compared to the placebo groups [53,57].

Based on the collected material, the intra-articular administration of local anesthetics does not seem to have any effect on the range of motion of the mandible. However, these conclusions are supported by only two clinical studies, which do not cover the first day [53,57]. 

#### 4.1.2. Ineligible Control Group Studies

In studies in which (a) the control differed not only in the absence of a local anesthetic or (b) in which all groups received an anesthetic, it was impossible to assess the impact of the substances in question on the treatment outcome. Local anesthetics were administered in combination with (1) hypertonic dextrose as a standard prolotherapy protocol (3 studies), (2) corticosteroid giving a worse effect than platelet-rich plasma without anesthetic (2 studies), or (3) sodium hyaluronate as a joint rinsing agent before viscosupplementation (2 studies). Lidocaine was also used for joint cavity rinsing before collecting synovial fluid for laboratory tests (1 study) [38,39,40,42,43,48,50,51].

#### 4.1.3. No Control Group Studies

Uncontrolled studies make it possible to determine changes in disease severity indicators during treatment, in relation to their initial values. However, they present difficulty in indicating which of the components of the therapy accounted for success. The following studies included combinations of local anesthetics with hypertonic dextrose and corticosteroids. In the hypertonic dextrose reports of Dasukil et al., Refai, and Zhou et al. it was unanimously assessed that prolotherapy brought the desired effect in the form of resolution of dislocations. The first two studies also presented a decrease in the intensity of pain during treatment [41,44,46].

The corticosteroid reports of Chakraborty et al. and Samiee et al. described intra-articular injections for treating post-hemimandibulectomy pain (single case) and mandibular immobility, respectively. In both situations, therapeutic success was achieved, expressed by pain relief and an increase in the range of mandible abduction, respectively [45,47].

The only two identified studies that describe the intra-articular administration of local anesthetics without therapeutically active additives are reports by Guarda Nardini et al. and Danzig et al. In the first of them, the physical administration of fluid under pressure into the joint cavity may have been of considerable importance, which in combination resulted in a significant increase in the range of jaw mobility and almost complete disappearance of articular pain in the context of other studies. The study of Danzig et al. showed immediate relief of pain after intra-articular administration of lidocaine in a group of 23 patients [49,52].

### 4.2. Potential Chondrotoxicity

The effect of intra-articular injections on the cartilage of the temporomandibular joints is currently an actively discussed topic [61]. Attempts are made to remove inflammatory mediators by performing arthrocentesis, introducing anti-inflammatory mediators in autologous blood concentrates, and autografting stem cells [13,62,63,64,65,66]. These interventions are chondroprotective and even regenerative in nature [67,68]. In the context of this state of advancement of injection techniques, the administration of substances with chondrotoxic potential seems to be unjustified.

The selection process inadvertently identified one experimental study on the administration of local anesthetics to the temporomandibular joint cavities. In a 2022 report by Asan et al., cytotoxicity of administration of 1 mL of lidocaine, bupivacaine, or articaine into the cavities of rabbit temporomandibular joints was indicated [69]. In the post-mortem examination, thinning and unevenness of articular cartilage and a reduced amount of collagen were observed compared to the placebo group receiving physiological saline solution. It was shown that the weakest adverse effects among the study groups were observed after the administration of articaine. The described results cannot be directly interpolated to humans due to the different body weights and volumes of joint cavities for the same volume (1 mL) of the agent administered in most of the identified clinical studies.

A 2023 report by Zhang et al. compiles data from various articles assessing the chondrotoxicity of local anesthetics (bupivacaine, ropivacaine, lidocaine, and mepivacaine) administered intra-articularly into shoulder and knee joints during arthroscopic surgery [70]. Joint chondrolysis was observed postoperatively due to significant disruption of chondrocyte cultures by local anesthetics. Bupivacaine toxicity was the highest due to its longest half-life among the tested local anesthetics; consequently, it is not recommended by Zhang et al. for intra-articular administration. Therefore, we call into question the safety of intra-articular administration of local anesthetics in temporomandibular disorders and encourage a review of experimental studies focused on this problem.

### 4.3. Limitations of the Evidence

The randomized controlled trials included in this review were characterized by high heterogeneity in terms of: (1) the type of local anesthetic used (bupivacaine, lidocaine, mepivacaine, and articaine); (2) number of intra-articular administrations (1 to 3 or as needed); (3) intervals between interventions (from 2 to 42 days); and (4) the total dose of the drug (from 2.5 to 40 mg).

### 4.4. Limitations of the Review Processes

The search query was based on English keywords, which made it impossible to identify and include fully foreign-language reports.

## 5. Conclusions

(1) Bupivacaine administered into the temporomandibular joint provided temporary pain relief, which lasted no longer than 24 h. In longer follow-ups, no statistically significant analgesic effectiveness was noted.
(2) There is no evidence of a statistically significant improvement in the range of jaw mobility after the intra-articular administration of local anesthetics.
(3) Local anesthetics administered intra-articularly have chondrotoxic potential, which requires verification in a separate systematic review.

## Figures and Tables

**Table 1:** Eligibility criteria.

	Criteria for Inclusion	Criteria for Exclusion
Problem	Patients diagnosed with temporomandibular disorders or healthy volunteers	Cadaver studies
Intervention	Local anesthetic intra-articular injection	None
Comparison	Injection with the omission or replacement of the local anesthetic	None
Outcomes	Articular pain severity or mandibular mobility range	Unquantifiable results
Settings	Randomized trials	Less than 5 patients per group

**Table 2:** Reports excluded at the eligibility stage.

First Author, Publication Year	Title	DOI or PMID Number (If the Former Was Not Assigned)	Reason for Exclusion
Bhargava, 2023 [38]	A Comparative Preliminary Randomized Clinical Study to Evaluate Heavy Bupivacaine Dextrose Prolotherapy (HDP) and Autologous Blood Injection (ABI) for Symptomatic Temporomandibular Joint Hypermobility Disorder.	10.1007/s12663-022-01738-x	Ineligible comparison
Shan, 2023 [39]	Platelet-rich plasma and hyaluronic acid for injection treatment of temporomandibular joint degeneration in Affiliated Stomatological Hospital of Guangzhou Medical University	10.57760/sciencedb.o00013.00022	Ineligible comparison
Prakash, 2022 [40]	Intra-articular platelet-rich plasma injection versus hydrocortisone with local anesthetic injections for temporomandibular disorders.	10.6026/97320630018991	Ineligible comparison
Dasukil, 2021 [41]	Efficacy of Prolotherapy in Temporomandibular Joint Disorders: An Exploratory Study.	10.1007/s12663-020-01328-9	No comparison
Louw, 2019 [42]	Treatment of Temporomandibular Dysfunction With Hypertonic Dextrose Injection (Prolotherapy): A Randomized Controlled Trial With Long-term Partial Crossover.	10.1016/j.mayocp.2018.07.023	Ineligible comparison
Gupta, 2018 [43]	Comparison between intra-articular platelet-rich plasma injection versus hydrocortisone with local anesthetic injections in temporomandibular disorders: A double-blind study.	10.4103/njms.njms_69_16	Ineligible comparison
Refai, 2017 [44]	Long-term therapeutic effects of dextrose prolotherapy in patients with hypermobility of the temporomandibular joint: a single-arm study with 1-4 year follow-up	10.1016/j.bjoms.2016.12.002	No comparison
Chakraborty, 2016 [45]	Ultrasound-Guided Temporomandibular Joint Injection for Chronic Posthemimandibulectomy Jaw Pain	10.1213/xaa.0000000000000384	No comparison (case report)
Zhou, 2014 [46]	Modified dextrose prolotherapy for recurrent temporomandibular joint dislocation.	10.1016/j.bjoms.2013.08.018	No comparison
Samiee, 2011 [47]	Temporomandibular joint injection with corticosteroid and local anesthetic for limited mouth opening.	10.2334/josnusd.53.321	No comparison
Refai, 2011 [48]	The efficacy of dextrose prolotherapy for temporomandibular joint hypermobility: A preliminary prospective, randomized, double-blind, placebo-controlled clinical trial	10.1016/j.joms.2011.02.128	Ineligible comparison
Guarda Nardini, 2002 [49]	Treatment of temporomandibular joint closed-lock using intra-articular injection of mepivacaine with immediate resolution durable in time (six months follow-up)	PMID: 11845117	No comparison
Sato, 1997 [50]	Effect of lavage with injection of sodium hyaluronate for patients with nonreducing disk displacement of the temporomandibular joint.	10.1016/s1079-2104(97)90337-1	Ineligible comparison
Kamada, 1993 [51]	Changes in synovial fluid N-acetyl-beta-glucosaminidase activity in the human temporomandibular joint with dysfunction.	PMID: 8182502	Ineligible comparison
Danzig, 1992 [52]	Effect of an anesthetic injected into the temporomandibular joint space in patients with TMD.	PMID: 1298765	No comparison

**Table 3:** Reports included in the systematic review.

First Author, Publication Year	Title	DOI or PMID Number (If the Former Was Not Assigned)
Zarate, 2020 [53]	Dextrose Prolotherapy Versus Lidocaine Injection for Temporomandibular Dysfunction: A Pragmatic Randomized Controlled Trial.	10.1089/acm.2020.0207
Ziegler, 2010 [54]	Analgesic effects of intra-articular morphine in patients with temporomandibular joint disorders: a prospective, double-blind, placebo-controlled clinical trial.	10.1016/j.joms.2009.04.049
Ayesh, 2007 [55]	Effects of local anesthetics on somatosensory function in the temporomandibular joint area.	10.1007/s00221-007-0893-4
Zuniga, 2007 [56]	The Analgesic Efficacy and Safety of Intra-Articular Morphine and Mepivacaine Following Temporomandibular Joint Arthroplasty	10.1016/j.joms.2007.04.001
Tjakkes, 2007 [57]	The effect of intra-articular injection of ultracain in the temporomandibular joint in patients with preauricular pain—A randomized prospective double-blind placebo-controlled crossover study	10.1097/ajp.0b013e31802f0950
Lobbezoo, 2003 [58]	Effects of TMJ anesthesia and jaw gape on jaw-stretch reflexes in humans	10.1016/s1388-2457(03)00155-x
Furst, 2001 [59]	The use of intra-articular opioids and bupivacaine for analgesia following temporomandibular joint arthroscopy: a prospective, randomized trial.	10.1053/joms.2001.25820
Gu, 1998 [60]	Visco-supplementation therapy in internal derangement of temporomandibular joint.	PMID: 11245058

**Table 4:** Study characteristics.

First Author	Diagnosis	Number of Patients (Study/Controls)	Local Anesthetic Type and Dose	Number of Injections/Interval (Days)	Total Anesthetic Dose	Interventions in Control Groups	Co-Interventions Common to the Study Group and Controls
Zarate	TMDs	15/14	0.2% lidocaine, 1 mL	3/28	6 mg	0.2% lidocaine + 20% dextrose, 1 mL injection	N/A
Ziegler	TMDs	12/36	0.5% bupivacaine, 2 mL	3/2	30 mg	(1) 2 mL of 0.9% saline, (2) 5 mg of morphine in X mL of 0.9% saline, or (3) 10 mg of morphine in X mL of 0.9% saline injection	N/A
Ayesh	Healthy volunteers	14/14 (contralateral control)	0.25% bupivacaine, 1 mL	1/N/A	2.5 mg	0.9% saline injection	N/A
Zuniga	Post-arthroplasty status	10/25	3% mepivacaine, 1 mL	1/N/A	30 mg	(1) 0.9% saline injection (placebo), (2) 0.1% morphine, 1 mL or (3) 0.1% morphine + 3% mepivacaine, 1 mL	N/A
Tjakkes	TMDs	20/20 (crossover control)	4% articaine + 1:200,000 pinephrine, 0.5 mL	2/14	40 mg	0.9% saline, 0.5 mL	Application of EMLA topical anesthesia 45 min before injection
Lobbezoo	Healthy volunteers	6/5	1% mepivacaine, 1 mL	2/14–42	20 mg	0.9% saline, 1 mL	N/A
Furst	Post-arthroscopy state	24/8	0.5% bupivacaine, 2 mL	1/N/A	10 mg	(1) 0.9% saline, 3 mL (2) 0.2% morphine, 1 mL or (3) 0.2% morphine, 1 mL + 0.5% bupivacaine, 2 mL	Postoperative application of morphine (4 mg, i.v.) and acetaminophen 325 mg + codeine 15 mg
Gu	TMDs	43/20	2% lidocaine, 1 mL	1 (more if needed)/N/A	20 mg	1% hyaluronic acid, 0.3–1 mL	Infiltration anesthesia of the preauricular area (2% lidocaine), articular cavity irrigation (0.9% saline, 5 mL)

TMDs—temporomandibular disorders; N/A—not applicable.

**Table 5:** Results of individual studies in the VAS/NRS articular pain domain over time. Values (with standard deviations where known) and mean differences (with standard errors where calculable) are provided.

First Author	Intervention Group	Sample Size	Initial	2 h	4 h	6 h	8 h	12 h	16 h	20 h	1 Day	2 Days	2 Weeks	1 Month	2 Months	3 Months	1 Year
Zarate	Lidocaine	21 joints	7.2 ± 0.8	N/S	N/S	N/S	N/S	N/S	N/S	N/S	N/S	N/S	N/S	5.4 ± 2.1 −1.8 ± 0.5 *	4.6 ± 2.2 −2.6 ± 0.5 *	4.3 ± 2.6 −2.9 ± 0.6 *	4.6 ± 2.5 −2.6 ± 0.6 *
	Lidocaine + dextrose	22 joints	7.2 ± 1.1	N/S	N/S	N/S	N/S	N/S	N/S	N/S	N/S	N/S	N/S	4.4 ± 2.4 −2.8 ± 0.6 *	4.4 ± 2.4 −2.8 ± 0.6 *	2.9 ± 2.6 −4.3 ± 0.6 *	2.4 ± 2.6 −4.8 ± 0.6 *
Ziegler	Bupivacaine 0.5%	12 joints	10	1.0 9.0	N/S	6.4 3.6	N/S	7.9 2.1	N/S	N/S	8.0 2.0	8.0 2.0	N/S	N/S	N/S	N/S	N/S
	Morphine 10 mg	12 joints	10	1.8 8.2	N/S	3.6 6.4	N/S	3.86.2	N/S	N/S	3.8 6.2	3.8 6.2	N/S	N/S	N/S	N/S	N/S
	Morphine 5 mg	12 joints	10	3.2 6.8	N/S	4.0 6.0	N/S	4.0 6.0	N/S	N/S	3.9 6.1	3.9 6.1	N/S	N/S	N/S	N/S	N/S
	Placebo (0.9% saline)	12 joints	10	6.2 3.8	N/S	7.1 2.9	N/S	8.1 1.9	N/S	N/S	8.5 1.5	8.51.5	N/S	N/S	N/S	N/S	N/S
Tjakkes	Articaine	20 patients	6.6 ± 2.3	N/S	N/S	N/S	N/S	N/S	N/S	N/S	N/S	N/S	5.3 ± 3.1 −1.3 ± 2.4 *	N/S	N/S	N/S	N/S
	Placebo (0.9% saline)	20 patients	5.8 ± 2.2	N/S	N/S	N/S	N/S	N/S	N/S	N/S	N/S	N/S	6.3 ± 2.7 0.5 ± 1.5 *	N/S	N/S	N/S	N/S
Furst	Bupivacaine	8 joints	N/S	2.2 ± 2.6 (baseline)	2.4 ± 1.9 0.2 ± 1.1 †	2.1 ± 2.2 −0.1 ± 1.2 †	1.3 ± 1.0 −0.9 ± 1.0 †	1.9 ± 1.4 −0.3 ± 1.0 †	2.8 ± 2.0 0.6 ± 1.2 †	2.1 ± 1.4 −0.1 ± 1.0 †	3.2 ± 3.2 1.0 ± 1.5 †	N/S	N/S	N/S	N/S	N/S	N/S
	Bupivacaine + morphine	8 joints	N/S	5.5 ± 2.9 (baseline)	6.8 ± 2.0 1.3 ± 1.2 †	7.2 ± 1.5 1.7 ± 1.2 †	6.2 ± 1.8 0.7 ± 1.2 †	4.8 ± 2.5 −0.7 ± 1.3 †	4.2 ± 2.4 −1.3 ± 1.3 †	4.1 ± 1.6 −1.4 ± 1.2 †	3.9 ± 1.4 −1.6 ± 1.1 †	N/S	N/S	N/S	N/S	N/S	N/S
	Morphine	8 joints	N/S	3.7 ± 2.4 (baseline)	3.4 ± 3.5 −0.3 ± 1.5 †	4.2 ± 3.6 0.5 ± 1.5 †	3.6 ± 2.0 −0.1 ± 1.1 †	3.8 ± 2.7 0.1 ± 1.3 †	4.8 ± 1.9 1.1 ± 1.1 †	2.7 ± 2.2 −1.0 ± 1.2 †	2.4 ± 2.1 −1.3 ± 1.1 †	N/S	N/S	N/S	N/S	N/S	N/S
	Placebo (0.9% saline)	8 joints	N/S	5.6 ± 1.9 (baseline)	7.2 ± 2.0 1.6 ± 1.0 †	6.8 ± 2.4 1.2 ± 1.1 †	7.6 ± 2.0 2.0 ± 1.0 †	6.7 ± 2.4 1.1 ± 1.1 †	4.7 ± 1.4 −0.9 ± 0.8 †	4.0 ± 2.6 −1.6 ± 1.1 †	4.4 ± 2.6 −1.2 ± 1.1 †	N/S	N/S	N/S	N/S	N/S	N/S

N/S—not specified; *—statistically significant (*p* < 0.05); †—no statistical significance (*p* ≥ 0.05).

**Table 6:** Results of individual studies in the mandibular abduction domain (in millimeters). Values (with standard deviations where known) and mean differences (with standard errors where calculable) are provided.

First Author	Intervention Group	Sample Size	Initial	2 Weeks	3 Months
Zarate	Lidocaine	14 patients	42.4 ± 9.3	N/S	47.8 ± 7.8 5.4 ± 3.2 †
	Lidocaine + dextrose	15 patients	38.7 ± 10.6	N/S	43.4 ± 9.8 4.7 ± 3.7 †
Tjakkes	Articaine	20 patients	N/S	N/S 2.0 ± 2.9	N/S
	Placebo (0.9% saline)	20 patients	N/S	N/S 0.4 ± 2.9	N/S

N/S—not specified; †—no statistical significance (*p* ≥ 0.05).

**Table 7:** Summary of findings.

Domain	Level of Evidence	Number of Studies	Total Sample Sizes in Local Anesthetic and Control Groups	Risk of Bias in Studies	Findings
Articular pain	Randomized controlled trials	4	61/40	From low to some concerns	- On the first day, bupivacaine provides a noticeably better analgesic effect than placebo. However, there is no data to prove the statistical significance of these differences. - In the period from 24 h to 2 weeks, there are no statistically significant differences between local anesthetics and the placebo. - No further observations were made with placebo control groups.
Mandibular mobility	Randomized controlled trials	2	34/20	From low to some concerns	- No statistically significant difference in jaw abduction was observed between the articaine and placebo groups 2 weeks after the intervention. - No statistically significant change in the range of mouth opening was observed after 3 months of observation of the group that received intra-articular lidocaine.

## Data Availability

All data are contained within the article.
